# Self-Management and Self-Efficacy in Patients With Acute Spinal Cord Injuries: Protocol for a Longitudinal Cohort Study

**DOI:** 10.2196/resprot.8054

**Published:** 2018-02-26

**Authors:** Tijn van Diemen, Eline WM Scholten, Ilse JW van Nes, SELF-SCI Group, Jan HB Geertzen, Marcel WM Post

**Affiliations:** ^1^ Sint Maartenskliniek Department of Rehabilitation Nijmegen Netherlands; ^2^ Center of Excellence in Rehabilitation Medicine Brain Center Rudolf Magnus University Medical Center Utrecht, Utrecht University and De Hoogstraat Rehabilitation Utrecht Netherlands; ^3^ University Medical Center Groningen Department of Rehabilitation Medicine, Center for Rehabilitation University of Groningen Groningen Netherlands

**Keywords:** spinal cord injuries, self-care, self-efficacy, rehabilitation, complications, social participation

## Abstract

**Background:**

People with recently acquired spinal cord injury (SCI) experience changes in physical, social and psychological aspects of their lives. In the last decades, attention has grown for aspects of self-management and self-efficacy in SCI research. However, we still do not know what the self-management and self-efficacy outcomes of first rehabilitation are and whether utilizing these skills may prevent secondary health conditions (SHCs) and increase participation and psychological adjustment early after SCI.

**Objective:**

To describe the course and determinants of self-management and self-efficacy during and after first SCI rehabilitation; and to determine theory-based associations between self-management and self-efficacy with SHCs, participation and psychological adjustment.

**Methods:**

Multicenter prospective longitudinal cohort study. All people with a newly acquired SCI admitted to one of the 8 specialized SCI rehabilitation centers in the Netherlands will be considered for inclusion in this study. Main assessments will take place during the first and last week of admission and 3, 6 and 12 months after discharge. The target sample is 250 participants. The primary outcomes are self-management (knowledge and execution of self-care) and self-efficacy (confidence in the ability to manage the consequences of SCI and of self-care). Secondary outcome measures are SHCs, participation and psychological adjustment to SCI.

**Results:**

The first results with the complete set of data are expected in June 2019.

**Conclusions:**

This protocol describes the SELF-SCI cohort study investigating self-management and self-efficacy of initial inpatient SCI rehabilitation. Second, associations will be investigated with SHCs, participation and psychological adjustment early after onset of SCI, until 1 year after discharge. The results will be used to test theories about motivation to perform health-promoting behaviors and adjustment to SCI.

## Introduction

### Overview

The global incidence of spinal cord injury (SCI) is estimated between 40 and 80 new cases per million population per annum [1]. In the Netherlands, between 400 and 500 people suffer SCI each year and the total number of persons living with SCI is estimated between 10.000 and 15.000 [2,3]. The primary loss of motor, sensory and autonomic function below the level of injury may lead to several secondary health conditions (SHCs) [4-7]. These primary and secondary consequences of SCI may affect the functional independence, participation and quality of life (QoL) of the person involved [8-10].

SHCs are common in people with SCI in the Netherlands [11], and their participation and QoL fall behind those of people without SCI [12,13]. One and 5 years after discharge from initial inpatient rehabilitation, many people with SCI reported urinary tract infections (57-59%), severe neuropathic pain (40-44%), pressure ulcers (29-46%), problematic spasticity (23-36%), and severe muscle or joint pain (22-35%) among other problems [14]. On the long term (>5 years post-injury), people with SCI report an average of 8 SHCs in the previous year [15], their participation in employment is lower compared to society as a whole [16], and more than a third experience mild to severe chronic mental health problems [9]. These findings are similar to results of studies in other countries [17-19].

The high prevalence and the chronic nature of SHCs, can lead to the conclusion that SCI should be seen as a chronic condition, rather than an incidental trauma. This also focuses attention to the crucial role and responsibility persons with SCI themselves have regarding the lifelong maintenance of their health and participation in the society. During first rehabilitation of people with SCI, learning and practicing self-management skills should therefore be a main concern.

Self-management is defined as the individual’s ability to manage the symptoms, treatment, physical and social consequences and lifestyle changes in accordance to a life with a chronic disease (Chronic Care Model) [20]. To be able to apply self-management, persons with SCI must have knowledge of their physical condition and how to prevent complications or control them if they do occur [18,21]. The high prevalence of SHCs reported in the SCI literature, however, suggest that at least part of the people with SCI lack sufficient self-management skills or do not use them properly [18,22].

Another concept associated with high prevalence of SHCs, especially psychological SHCs, is self-efficacy [23]. Self-efficacy is defined as the belief that one can successfully execute the behavior required to produce the desired outcomes [24]. Negative associations are found between self-efficacy and depression and anxiety. The negative association between self-efficacy and the occurrence of physical SHCs of people with SCI is still unclear [23]. There is, to date, also limited information about the course of self-efficacy and self-management during and after the SCI rehabilitation. Nor do we know if self-management and self-efficacy may prevent SHCs from occurring.

The SELF-SCI study has been designed to investigate this gap. The aims of the SELF-SCI study are: 1) to describe the course of self-management and self-efficacy during and after the first year of clinical SCI-rehabilitation; 2) to examine determinants of self-management based on the theory of planned behavior (TPB); 3) to examine determinants of adjustment after SCI based on the SCI adjustment model (SCIAM).

### Theoretical Background

To understand how people handle the consequences of their SCI, it is not only important to know the aspects involved in health-related behavior, but also the way people adjust to this situation. Therefore we will use two complementary models; the Theory of Planned Behavior (TPB) which has its focus on health-promoting behavior [25], and the SCI adjustment model (SCIAM) [26] which describes the way people adjust after SCI.

**Figure 1:**
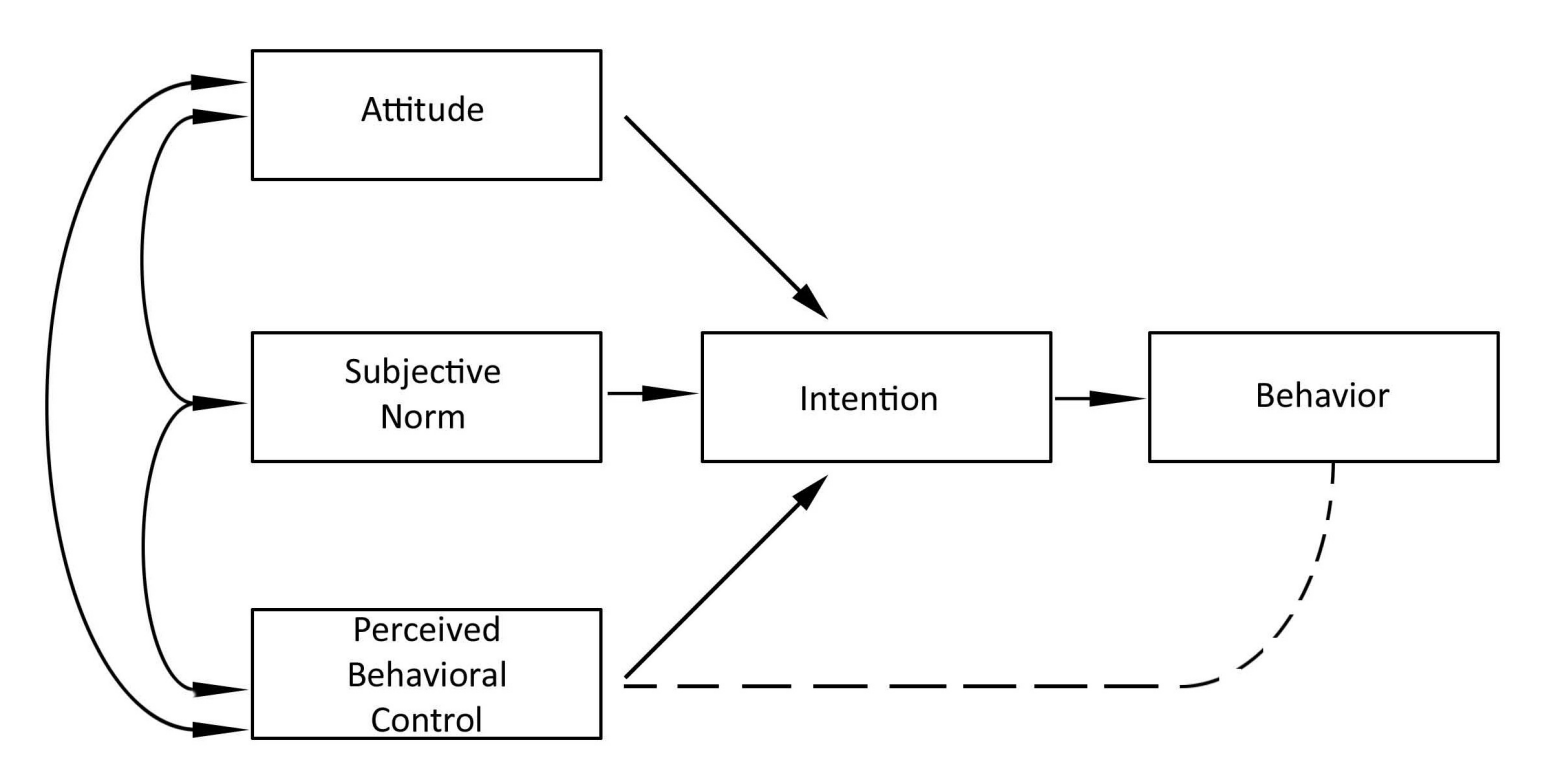
Scheme of theory of planned behavior.

According to TPB, the intention of people to perform health-promoting behaviors depends on their attitude, subjective norms and perceived behavior control. The scheme of TPB is depicted in Figure 1. Attitude is the individual's prospective evaluation of self-performance of a particular behavior [25]. Subjective norm refers to the perceived social pressure to perform certain behavior [25]. Perceived behavioral control refers to an individual’s belief in their ability to succeed in specific situations or accomplish a task, also called self-efficacy [25,27].

**Figure 2:**
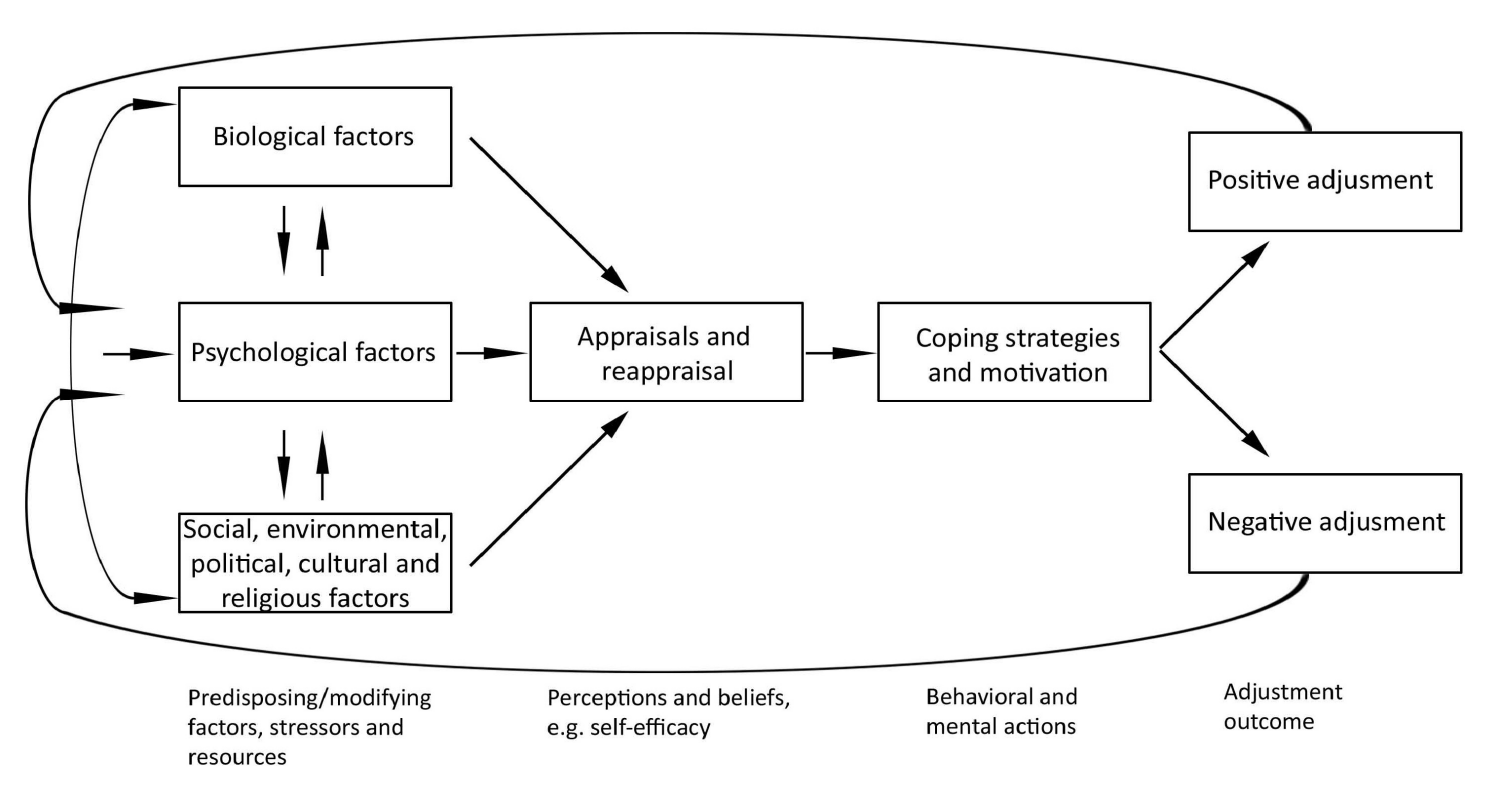
Scheme of spinal cord injury adjustment model.

The SCIAM (Figure 2) [26] is based on the notion that adjustment to SCI is influenced by physical aspects, psychological resources and social factors. These aspects interact with each other and influence the person’s appraisal of their situation. This will lead to certain ways of coping and levels of motivation. The result will be positive or negative adjustment. Adjustment has a psychological component, reflected in well-being or distress, and a social component, reflected in social engagement/participation.

The continuous process of appraisal and re-appraisal of the situation has a central role within SCIAM. First there is the perception of the current situation, the primary appraisal, then there is the secondary appraisal to what extent the person has sufficient resources to deal with this situation. These beliefs are influenced by the aforementioned physical, social and psychological factors. A variety of psychological resources have been associated with adjustment in the literature [12,28]. Resources with a high potential to predict adjustment and with a minimum of conceptual overlap are: self-efficacy, resilience, personality and meaning in life [12].

In studies on self-efficacy during and shortly after SCI rehabilitation, moderate relationships between self-efficacy with participation and psychological wellbeing were found [23,29,30]. In the chronic stage, moderate to strong relationships between self-efficacy with adjustment variables (especially depression and anxiety) were found [23,31,32]. Self-efficacy can be conceptualized and measured at different levels [23]. General self-efficacy (GSE) refers to the self-beliefs of a person to cope with a variety of difficult commands in general [27,33]. Disability management self-efficacy (DMSE) is defined as the confidence that people have in their ability to manage the consequences of their chronic condition [34]. Finally, self-care self-efficacy (SCSE) refers to specific beliefs concerning the opportunities to perform appropriate self-care. The specific self-efficacy described within TPB is best categorized at the level of SCSE. The secondary appraisal process in SCIAM is self-efficacy at the level of DMSE. GSE, finally, is considered to be a trait variable that will not change much over time, and therefore is seen as one of the psychological resources as described in SCIAM. DMSE and SCSE are seen as state variables that are more situation-specific and vary over time. From literature as well as from a theoretical point of view self-efficacy seems to play an important role in participation and psychological adjustment.

To investigate the role of both self-management and self-efficacy, TPB and SCIAM were used to design the current study. All the aspects described in both theoretical models were taken into account by measuring each aspect through one or more assessment tools.

## Methods

### Overview

SELF-SCI is a multicenter prospective longitudinal cohort study during the first SCI inpatient rehabilitation until one year after discharge. To describe the course of self-management and self-efficacy, repeated measures of the main outcome variables are used. In this quantitative study all aspects described in the theoretical models (TPB and SCIAM) are investigated, to examine determinants of self-management and adjustment after SCI.

### Data Collection tools

#### First Aim

The main outcome variables of the first aim of this study are self-management and self-efficacy. Self-management is operationalized as knowledge and execution of self-care. Selfefficacy is measured at two levels; the level SCSE and of DMSE.

*Self-management* will be measured with a questionnaire concerning the knowledge and execution of self-care. The 13 questions about the knowledge of self-care can be answered on a 5-point scale ranging from certainly not true to certainly true. An example of a question is: “I know what to do when confronted with a pressure ulcer.” The 14 questions about the execution of self-care can be answered on a 4-point scale ranging from never to always. An example of a question is: “I maintain my physical fitness as good as possible.” This list was previously used among community-dwelling people with SCI, with a high internal consistency α=.80 [35]. Because knowledge and execution of self-care must be acquired during rehabilitation, this questionnaire is administered for the first time at discharge.

*Self-care self-efficacy* will be measured with the Managing Disease in General subscale of the Self-efficacy for Managing Chronic Disease Scale [36]. This subscale consists of 5 items with a 0-10 numeric rating scale (NRS) which indicate to what degree participants have confidence in the asked behavior or judgment. The internal consistency is high α=.87 [36]. Some questions have been adapted to get a better fit with the research question. An example of a question is: “How confident are you that you can do all the things necessary to manage your condition on a regular basis?”

*Disease management self-efficacy* will be measured with the short version of the University of Washington Self-efficacy Scale [34]. This 6-item version has a 5-point scale ranging from not at all confident to totally confident. This scale has been validated for people with SCI and multiple sclerosis [34,37]. The internal consistency of the short version is high (α=.90) [34]. At admission one question will be added concerning the confidence one has about the increase of DMSE during rehabilitation on a 0-10 numeric rating scale (NRS).

#### Second Aim

The main outcome variable of the second aim is self-management. Main determinants of self-management are SCSE, attitudes towards self-management and subjective norm.

*Attitude* to perform health behavior in SCI was, to our knowledge, not studied previously. A new scale was constructed, the Motivation for Health Care Scale. Based on the theoretical background of TPB a total of 6 questions were formulated, covering the subject of attitude to perform health behavior in people with SCI. On each question the participants can point out to what extent the given health behavior is important to them on a 0-10 NRS. An example of a question is: “Do you find it important to have an active role in preventing health problems?” Data of the current study will be used to investigate reliability and convergent validity of this scale.

*Subjective norm* is operationalized as experienced stimulation from the people close to the participant, with respect to self-care. While no such scale existed, a new scale was constructed for this purpose; the Stimulation to Perform Self-care List. On 6 questions with a 0-10 NRS, the participants can state to what extent they are stimulated to perform self-care and health-promoting behaviors by people in their social environment (eg, “My partner/family stimulate me to take good care for myself?”).

#### Third Aim

The main outcome variable of the third aim is adjustment. Adjustment is operationalized as distress, illness cognitions, life satisfaction and participation. Demographic, physical -, social aspects and psychological resources are taken into account as determinants of adjustment.

*Distress* will be assessed using the Hospital Anxiety and Depression Scale [38]. This scale is a commonly used measure of distress and contains 14 statements equally divided in two scales; Depressive mood and Anxiety. Participants will be asked to indicate the extent to which they agree with each item, on a 4-point scale [38,39].

*Illness cognitions* will be assessed using an adapted version of the Illness Cognitions Questionnaire [40,41]. This instrument contains 18 statements divided into three subscales: Helplessness, which measures the aversive cognitive attributions attached to the SCI; Acceptance, which measures neutralizing connotations of the condition; and Disease benefits, which measures the positive meaning given to the SCI. Participants will be asked to indicate the extent to which they agree with each statement, ranging from 1 (not at all) to 4 (completely).

*Life Satisfaction* will be assessed using 2 Life Satisfaction questions [42]: one question about the QoL at this moment with 6 answer categories (ranging from very unsatisfying to very satisfying), and the second question about the comparison of QoL now with the QoL before the SCI with 7 answer categories (ranging from much worse to much better) [43].

*Participation* will be measured using the Utrecht Scale for Evaluation of Rehabilitation-P, participation [44,45]. The scope of this 32-question scale is to investigate the frequency of participation in daily activities, experienced participation restrictions due to the SCI and satisfaction with participation. At T1 the questions will somewhat be changed to assess the activity level and the satisfaction with these activities before the SCI, as has been done before [46]. One year after discharge the original scale will be used.

*Participation* will further be assessed using two questions from the Craig Handicap Assessment and Reporting Technique [47]. These two questions (how many hours a day one is out of bed and how many days per week one gets out of the house) are more often used for this purpose [48].

### Determinants of Adjustment

*SCI characteristics* (time since injury; cause of the lesion: divided into traumatic and non-traumatic; level and severity of injury according to the International Standards for Neurological Classification of Spinal Cord Injury) [49] will be determined by a trained rehabilitation physician at admission and discharge.

*Functional independence* in self-care and mobility will be measured with the corresponding subscales of the Utrecht Scale for Evaluation of Rehabilitation [50]. This observation scale consists of 7 items for each subscale, that can be scored by a professional on a 5-point scale. Higher scores indicate higher independence.

*Experienced pain and fatigue* during the past week will be measured with a NRS ranging from 0-10.

*Medical consumption* will be measured with questions about the amount of visits to health professionals like physicians, physiotherapists, stay in a hospital and the amount of help from family and friends for the past three months. Other questions will be about the occurrence of medical complications: pressure ulcers, incontinence, urinary tract infections and weight gain or loss.

*Influence of SHCs* will be measured with the Spinal Cord Injury Secondary Conditions Scale [51]. From the original 16 items, 12 were selected, which can be influenced by the participant with health-promoting behaviors. The participants have to rate on a 4-point scale how much each health problem affected their activities and independence in the last three months [51].

The *appraisal of the current situation* will be measured with the Appraisal Life Events Scale [52]. Using 16 adjectives, participants will respond how they appraised their life in the past 3 months on a 6-point scale. The Appraisal Life Events Scale is recently used in a study with community-dwelling people with SCI [32].

The *general self-efficacy* will be measured with the General Competence Scale, the ALCOS-12, the Dutch version of the General Efficacy Scale from Sherer [33]. The ALCOS-12 consists of 12 questions with a 5-point scale, concerning the confidence to solve problems in general.

*Resilience* will be measured with the Brief Connor-Davidson Resilience Scale. This short version consists of 10 items with a 5-point scale [53,54].

*Personality* will be measured with the subscale neuroticism of the Eysenck Personality Questionnaire [55]. This scale consists of 12 dichotomous questions. Neuroticism has a strong association with QoL according a systematic review [12].

*Meaning in life* will be measured with the short version of the Purpose in Life Scale [56]. This scale consists of 4 of the original 20 questions that can be answered on a 7-point NRS.

*Coping* is operationalized in two different ways, previously be proven to be of influence on adjustment in people post-stroke [57], namely passive coping and proactive coping.

To measure the *passive coping,* the passive reaction pattern subscale of the Utrecht Coping List will be used [58]. This subscale consists of 7 questions with a 4-point scale.

The *proactive coping* style will be measured by the Utrecht Pro-active Coping Competence Scale short version [59]. This scale measures to what extent the participant is proficient to anticipate on difficult situations in the future on a 4-point scale. This short version, consisting of 7 of the original 21 items, is recently developed and had a high internal consistency (α=.90) and a very high intra class correlation (=.96) with the total list (Post in preparation).

*Social support* will be assessed by the Social Support List-12 [60]. This short version consists of 12 items with a 4-point Likert scale. There are three sub-scales; everyday social support, support in problem situations and esteem support [60,61].

The way participants are *empowered* during the rehabilitation phase will be measured with a selection of questions from the Patient Assessment of Chronic Illness Care [62]. These 8 questions reflect the way in which the participants are involved in decision making during the rehabilitation phase. On a 5-point scale, participants can respond to what extent they were supported by the professionals, in making their own decisions and to perform self-care, during clinical rehabilitation [62].

*Demographic variables* including age, sex, living with a partner, and educational level will be assessed.

An overview of all measurement instruments is shown in Table 1.

### Ethical Considerations

The Medical Ethics Committee of the University Medical Centre Utrecht declared that this protocol does not need formal ethical approval under the Dutch law regulating medical research in human beings (reference number: 15-449/C). The Medical Ethics Committees of all participating rehabilitation centers approved this protocol. The study will be carried out according to the code of conducts formulated by Helsinki code. As part of this code all participants will give written informed consent before entering the study.

**Table 1:** Measurement instruments on the different test occasions.

Outcome measures		Instrument	T1	T2-T4	T5	T6	T7	T8
**Primary outcome measures**								
	Self-management (first and second aim)	Knowledge and execution of self-care			X			X
	Self-care Self-efficacy (first aim)	Self-efficacy for Managing Chronic Disease Scale, Managing disease in General subscale			X			X
	Disability management Self-efficacy (first aim)	University of Washington Self-Efficacy Scale-Short Form	X		X	X	X	X
	Distress (third aim)	Hospital Anxiety and Depression Scale	X		X			X
	Illness cognitions	Illness Cognitions Questionnaire	X		X			X
	Life satisfaction (third aim)	Two Life Satisfaction questions	X	X	X	X	X	X
	Participation (third aim)	Utrecht Scale for Evaluation of Rehabilitation, participation part	X					X
	Participation (third aim)	Craig Handicap Assessment and Reporting Technique, 2 questions				X	X	X
**Determinants of second aim**								
	Stimulation from environment	Stimulation to Perform Self-Care List			X			X
	Motivation to prevent health problems	Motivation for Health Care List			X	X	X	X
**Determinants of third aim: Biological and functional determinants**							
	SCI characteristics	—	X		X			
	Functional independence	Utrecht Scale for Evaluation of Rehabilitation	X		X			
	Experienced pain, fatigue and mood	Numeric Rating Scale	X	X	X	X	X	X
	Medical consumption	Questions about received help			X	X	X	X
	Experienced complications	Spinal Cord Injury Secondary Conditions Scale			X	X	X	X
**Determinants of third aim: Psychological determinants**								
	Appraisal	Appraisal Life Events Scale	X		X			X
	General self-efficacy	General Competence Scale (ALCOS-12)	X					
	Resilience	Brief Connor-Davidson Resilience Scale	X					
	Personality	Eysenck Personality Questionnaire, neuroticism subscale	X					
	Meaning in life	Purpose in Life Scale (short version)	X					
	Passive coping	Utrecht Coping List, passive reaction patron subscale	X					
	Active coping	Utrecht Pro-active Coping Competence Scale (short version)	X					
**Determinants of third aim: Social determinants**							
	Social support	Social Support List-12	X					
	Demographic determinants	Age, sex, level of education, marital status, and other	X					

### Study Setting and Participants

In the Netherlands 8 rehabilitation centers are specialized in SCI rehabilitation. All 8 centers participate in this study. In this protocol patients are eligible for this study if they have been admitted for inpatient rehabilitation with a clinically confirmed diagnosis of SCI, this is their first inpatient rehabilitation after the onset of the SCI, and this admission will last for at least 4 weeks. Furthermore the patient must be at least 18 years old and be able (with help if necessary due to hand function problems) to complete the self-report questionnaires. Patients with severe cognitive problems are excluded, as well as patients who have insufficient knowledge of the Dutch language to understand and complete the questionnaires. Patients are also excluded from this study if they have a limited life expectancy, for example in case of cancer-related SCI. There are no restrictions regarding the severity of SCI or maximum age. Decision on in/exclusion is based on the clinical judgment by the rehabilitation physician and will be checked by the research assistant. If the participants are not able to complete the questionnaire because of hand function problems, help is offered by a research assistant.

All eligible patients will be informed about the study by their rehabilitation physician on the first day of admission into rehabilitation. One or two days later the research assistant will inform the patient more extensively. After informed consent is given, the research assistant will provide the participant with the first comprehensive questionnaire (T1). Next, a short 5-item questionnaire will be administered after 4 (T2), 8 (T3) and 12 weeks (T4), if at that time the participant is still admitted for at least two weeks. In the last week of admission the second comprehensive questionnaire (T5) will be administered. Three (T6) and six months (T7) after discharge a brief questionnaire will be sent to the participants, and one year after discharge the final comprehensive questionnaire (T8) will follow. During inpatient rehabilitation, participants will complete paper/pencil versions of the questionnaires. After discharge, the participants can choose whether they want to complete the questionnaire on paper or online (NetQ package). Before the questionnaire is sent after discharge (T6 to T8), the participants will be contacted by phone, further two reminders will be send in case of no response. Participants will not be offered monetary or non-monetary compensation for their efforts.

A total of 250 participants will be recruited. This target number is chosen to allow regression models with 15 determinants with sufficient statistical power per determinant in the model. An estimated 350-400 people who fit the in- and exclusion criteria are admitted to one of these 8 specialized centers each year. Therefore, it seems feasible to include the desired 250 participants within the two-year inclusion period from January 2016 until December 2017.

### Data Analysis

All data will be entered into SPSS statistical program for Windows (version 24). The manually entered data will be checked by a second person. The data from the online questionnaires will be exported and merged with the manually entered data. When all data is entered descriptive statistics will be performed. Outliers and scores out of range of the questionnaires will be double-checked. Next, multilevel analysis, with mixed methods approach, will be performed to estimate differences between the three major assessments (T1, T5 and T8) and between all 8 assessments with a limited number of variables. Next, latent class growth mixture modeling will be used to investigate if there are different trajectories of self-management and DMSE between admission and one year after discharge. Prediction of problems regarding self-management and DMSE on T8 will be analyzed using multivariate regression models. Also relationships between self-management, DMSE and SCSE on the one hand and SHCs, participation and psychological adjustment on the other will be analyzed using multivariate regression analyses and path analysis.

The first aim of this study is to describe the course of self-management and self-efficacy during the first SCI rehabilitation period until one year after discharge. All available data concerning the three main variables will be used. For the second and third aim (examine the determinants of self-management and adjustment) the theory will be tested using a path analysis.

## Discussion

The SELF-SCI Cohort study investigates the changes in self-management and self-efficacy of people with a recently acquired SCI during the first initial rehabilitation until one year after discharge. Next, this study determines, based on theories about motivation to perform health-promoting behaviors and adjustment to SCI, to what extent self-management, DMSE and SCSE are predictors of SHCs, participation and psychological adjustment.

There are several reasons why this cohort study is innovative. First its focus on the changes in self-management, self-efficacy over time, from shortly after the occurrence of SCI until one year after inpatient rehabilitation. Traditionally, much research and rehabilitation care has focused on the physical and functional impact of SCI. Research on psychological impact of SCI is most often cross sectional and performed in community-dwelling people with SCI. In addition, this longitudinal study focuses on the post-acute phase until one year after SCI-rehabilitation. Second, this study will investigate the relationship between self-management and self-efficacy on the one hand and SHCs, participation and psychological adjustment on the other. With a growing amount of older people with SCI, these SHCs and reduced participation in society is of major interest for health workers and policy makers. Thirdly, this study is theory driven. The present study will extensively investigates the influence of motivation to perform health-promoting behaviors and adjustment to SCI on self-management and self-efficacy. All the variables within both theories will be taken into account, as much as possible, in order to be able to test these models for the SCI population [31,32]. And lastly, this is a nation-wide study including all 8 rehabilitation centers with a SCI specialization in the Netherlands. This means that a broad range of people, who are recently confronted with SCI, including people with traumatic and non-traumatic SCI and irrespective of age and severity of SCI, will be included in this study.

A limitation of this study could be the fact that the outcomes are only measured with self-assessment questionnaires. However, we do not consider this as a problem, because especially DMSE and SCSE are subjective concepts which we will measure with a validated scale.

In conclusion, the information which will be gathered in the present study, especially about the influence of self-management and DMSE on SHCs and participation, will be used to establish better rehabilitation care and to develop new interventions for SCI patients. This should allow people with SCI to make optimal use of their capacity to deal with their new situation.

## Abbreviations

DMSE: disability management self-efficacy
GSE: general self-efficacy
NRS: numeric rating scale
QoL: quality of life
SCH: secondary health condition
SCI: spinal cord injury
SCIAM: spinal cord injury adjustment model
SCSE: self-care self-efficacy
TPB: theory of planned behavior
